# Supplementary material for: The case for decreased surgeon-reported complications due to surgical volume and fellowship status in the treatment of geriatric hip fracture: An analysis of the ABOS database
Source: PLoS One. 2022 Feb 25;17(2):e0263475. doi: 10.1371/journal.pone.0263475 (PMC8880652; doi:10.1371/journal.pone.0263475)
Supplement: Attachment — Submitted filename: Review_PLOS_ONE.docx [file pone.0263475.s001.docx]

Dear Editor,

It was a pleasure for me to review the above-mentioned manuscript for PLOS-ONE.

**AIM OF THE STUDY**

The aim of this study is important as the final goal of any fellowship training (providing better care to a defined sub-population of orthopaedic patients) still needs to be scientifically investigated, at least for trauma patients. Right now, fellowship is recognized as the “right to operate on some patients” by academics, hospital/clinic administrators and sometimes even by patients, but this “right” relies on few objective data.

**LEVEL OF ENGLISH AND EDITION**

Minimal corrections needed. See one of my comments.

**QUALITY OF THE STUDY/MANUSCRIPT**

-General:

On the Plos One Editorial Manager page, corresponding author is Jonathan N Grauer, whereas on the title page, corresponding author is Taylor D Ottesen. Please correct one of these statements to be consistent throughout the manuscript.

-Abstract:

-Lines 47-48: “no prior study has investigated…”; however, in the Introduction, the authors cite references 5 & 23 (lines 114-117). Please modify the abstract, stating something like “Rare studies…” or “Scarce studies…” or something else.

-Line 61: after reading the Methods and the Results chapters, “1994-2004” should be replaced by “1999-2004”. Also see my comments on percentages (9.43% -> 6.8%, 60.92% -> 22%) on Results/Table 1.

-Manuscript

-Introduction:

-Line 95: replace “including” by “includes”.

-Lines 120-121: the statement should be mitigated, as references 5 & 23 studied this point.

-Materials and methods:

-Line 149: define the abbreviation “CPT” at its first appearance (line 149), not at its second appearance (Line 158).

-“Data” section: please define which states are included in each of the 6 regions. This might be obvious for US (and maybe Canadian) readers, but not for the rest of the world. As a non-US reviewer, I am for instance interested in incongruous/off-the-grid questions, such as: to which region belong Alaska and Hawaii? Were data from Puerto Rico, US Virgin Islands and other overseas territories included? This could be described in a Figure or a Table to save word count.

-“Data” section: “Number of cases completed by the candidate during…”. Does this number really reflect the volume? Might underestimation be possible? Please add a short statement about this issue, either in the M&M chapter, or in the limitation section.

Results:

-I am a little bit surprised by the fact that the tiny differences in ages (82.2 +/- 8.2 vs. 82.8 +/- 7.8 vs. 82.6 +/- 7.8, Line 199) and gender repartition (71,6% female vs. 73.2% vs. 73.5%, Line 201) among different fellowship groups reaches statistical significance which such mighty p-values. I would simply ask the authors to double-check these results (this also applies to Table 1).

-Lines 201-204/Table 1:

1° see my comment on abstract Line 61.

2° I think percentages in Table 1 should be corrected. For example: In years 1999-2004, 14954 procedures were performed (1016 + 1894 + 12044), meaning that Trauma fellowship trained surgeons performed 6.8% of the procedures (1016 out of 14954, not 1016 out of 10796), Adult reco 12.7% of the procedures (1894 out of 14954) and others 80.5% (12044 out of 14954). For years 2005-2010, my calculations result in 11.1%, 14% and 74.9% respectively. For years 2011-2016, 22%, 16.3% and 61.7% respectively; P-value should thus be recalculated, but the interpretative text is probably still ok.

-Table 1:

1° I think after calculation that N for “Other” should be 51910 rather than 60612. However, percentages for the whole column seem to be generally correct:

Total N for Sex/Other, Year/Other and Procedure type/Other is 51910-ok;

Total N for Region/Other is 51643-please correct or add a line for “n.a. region” or “other region”.

P-values might have to be corrected.

2° Total N for Region/Trauma is 10710 and does not correspond to 10769: please correct or add a line for “n.a. region” or “other region”.

3° Total N for Region/Adult reco is 10732 and does not correspond to 10748: please correct or add a line for “n.a. region” or “other region”.

-Table 2: I think after calculation that N for other should be 51910 rather than 60612. However, percentages for the whole column seem to be correct. P-values might have to be corrected.

-Tables 3a and 3b: I think after calculation that N for other should be 51910 rather than 60612. However, percentages for the whole column seem to be correct. P-values might have to be corrected.

Discussion:

-As long as corrections following my remarks on the Results section and Tables do not provoke unexpected changes in the interpretation of results, this section won’t need any corrections to be performed.
